# Application of Negative Curvature Hollow-Core Fiber in an Optical Fiber Sensor Setup for Multiphoton Spectroscopy

**DOI:** 10.3390/s17102278

**Published:** 2017-10-06

**Authors:** Maciej Andrzej Popenda, Hanna Izabela Stawska, Leszek Mateusz Mazur, Konrad Jakubowski, Alexey Kosolapov, Anton Kolyadin, Elżbieta Bereś-Pawlik

**Affiliations:** 1 Department of Telecommunications and Teleinformatics, Wroclaw University of Science and Technology, 50-370 Wroclaw, Poland; maciej.popenda@pwr.edu.pl (M.A.P.); hanna.stawska@pwr.edu.pl (H.I.S.); 2 Advanced Materials Engineering and Modelling Group, Wroclaw University of Science and Technology, 50-370 Wroclaw, Poland; leszek.mazur@pwr.edu.pl (L.M.M.); kudzu.jakubowski@gmail.com (K.J.); 3 Fiber Optics Research Center of Russian Academy of Sciences, Moscow 119333, Russia; kaf@fo.gpi.ru (A.K.); kolyadin@fo.gpi.ru (A.K.)

**Keywords:** negative curvature fiber, hollow core fiber, multiphoton fluorescence, photonic crystal fiber sensor

## Abstract

In this paper, an application of negative curvature hollow core fiber (NCHCF) in an all-fiber, multiphoton fluorescence sensor setup is presented. The dispersion parameter (D) of this fiber does not exceed the value of 5 ps/nm × km across the optical spectrum of (680–750) nm, making it well suited for the purpose of multiphoton excitation of biological fluorophores. Employing 1.5 m of this fiber in a simple, all-fiber sensor setup allows us to perform multiphoton experiments without any dispersion compensation methods. Multiphoton excitation of nicotinamide adenine dinucleotide (NADH) and flavin adenine dinucleotide (FAD) with this fiber shows a 6- and 9-fold increase, respectively, in the total fluorescence signal collected when compared with the commercial solution in the form of a hollow-core photonic band gap fiber (HCPBF). To the author’s best knowledge, this is the first time an NCHCF was used in an optical-fiber sensor setup for multiphoton fluorescence experiments.

## 1. Introduction

Multiphoton microscopy, first presented by Webb et al. [1], has become a standard imaging procedure for many laboratories, as it allows for greater imaging depths and reduced biological phototoxicity when compared to single-photon methods. Other non-linear optical phenomena (NLOP), such as Second Harmonic Generation (SHG) or Coherent Anti-Stokes Raman Scattering (CARS), are also being implemented in the imaging systems, forming powerful diagnostic tools [2,3,4,5,6]. Ultrashort, high-energy laser pulses are essential for efficient induction of the NLOP, and the transmission of such pulses through optical fibers is problematic because of the pulse’s temporal broadening due to the dispersion. Currently, this problem can be addressed in two ways—either by using dispersion compensation systems [7,8,9] or by using hollow-core, photonic bandgap fibers (HCPBF’s) [10], or both [11]. However, dispersion compensating systems are bulky setups, while HCPBF’s keep their dispersion parameters only within relatively narrow optical bandwidths, which becomes a problem when considering the optical spectrum of two photon absorption cross sections of biological fluorophores [12].

Negative curvature hollow-core fibers (NCHCF) are a relatively new type of microstructured hollow-core fibers, guiding the light in their air-filled core. They are significantly different from HCPBFs—their optical structure is much simpler, and the ARROW (Antiresonant Reflecting Optical Waveguides) guiding mechanism [13] allows their use at different optical bandwidths, usually very distant from each other [14,15]. Additionally, because of a very low coupling of core and cladding modes, as well as their hollow-core architecture, they are a promising possibility for the transmission of high-power, ultra-short optical pulses [16,17]. Up to the present day, Sherlock et al. [18] were the first and only ones to present the potential of this type of fiber in multiphoton microscopy. In this paper, we present an application of NCHCF fibers in an optical fiber sensor setup for the purpose of multiphoton fluorescence experiments. By creating a simple fiber probe setup we were able to perform a multiphoton excitation (MPE) of two endogenous fluorophores—NADH and FAD—and collect their fluorescence spectra. Unique dispersion parameters of the NCHCF occur in the range of 680–750 nm [19], which is optimal for efficient excitation of the aforementioned fluorophores. When comparing the suitability of two different fibers — NCHCF and HCPBF — a large increase in the total fluorescence intensity was observed when using the NCHCF as the excitation fiber.

## 2. Hollow-Core Fibers

**Figure 1:**
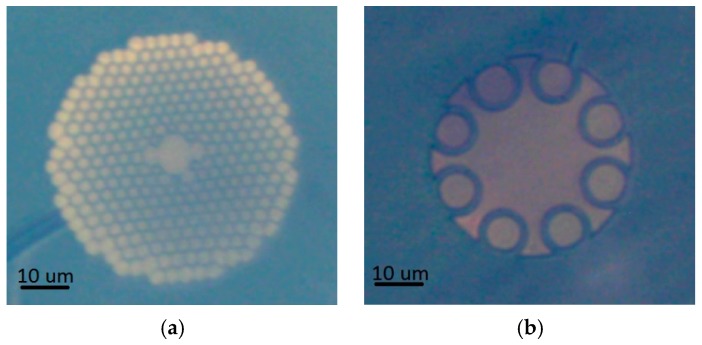
Cross sections of microstructured, photonic bandgap fiber—HC-800-02 from NKT Photonics (**a**) and NCHCF with eight separated capillaries (**b**). An approximate scale bar of 10 μm is presented in the bottom left corner of each picture. Central part of each fiber is its core, surrounded with either a large number of small air holes (HC-800-02, (**a**)) or a single row of larger capillaries (NCHCF, (**b**)).

Two different types of optical fibers were used during this research for the purpose of femtosecond pulse delivery—the custom-made NCHCF, which was previously characterized in [19], and a commercial HCPBF—the HC-800-02 (NKT Photonics, Birkerød, Denmark). The cross sections of both fibers are presented in Figure 1.

HC-800-02 has a microstructured cladding consisting of a large number of small air holes, which creates a photonic band gap across its operating spectrum, effectively trapping the light inside its air-filled core. According to this fiber specification [20], its core diameter is 7.5 μm, while its D (dispersion parameter) values range from −100 ps/nm × km at 760 nm to ~200 ps/nm × km at 870 nm, crossing zero at ~775 nm. The other fiber used —NCHCF—contains a microstructured cladding as well. However, this cladding construction is much simpler as it consists of only one row of large (when compared to the used HCPBF) capillaries. Light in this type of fibers propagates along the core walls due to the mechanisms described by the ARROW model. The core of this fiber is surrounded by eight separate capillaries, creating the negative curvature condition. The diameter of the core is 21 μm, while the capillaries have their walls 828 nm thick, according to [19]. The HC-800-02 has already proven useful for the multiphoton experiments [11], while the presented NCHCF has not been applied for this purpose so far.

## 3. Multiphoton Fluorescence Sensor Design and Construction

**Figure 2:**
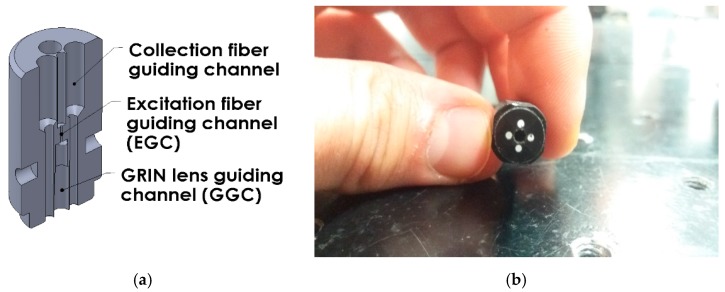
Fiber sensor head cross section (**a)** and a photograph of the sensor detection face (**b**). In the picture of the sensor face, the central part is a hole for the GRIN lens, while the four smaller circles surrounding it are collection fiber faces. The sensor’s outer diameter is 10 ± 0,05 mm. Sensors head was fabricated in the university CNC (Computerized Numerical Control) center.

A schematic cross section and a photograph of the sensor head are presented in Figure 2. Although the idea of this type of sensor has already been presented by many other researchers [21,22,23,24,25,26], never before has it been used with a combination of two types of fibers so distinct from each other—hollow core fibers and classic, solid core ones.

The central part of the sensor consists of two concentric channels—a GRIN lens guiding channel (GGC) and an excitation fiber guiding channel (EGC), with diameters of 1.8 mm and 0.25 mm, respectively. Channels were drilled at the two opposite planes, parallel to the sensor’s main axis. This type of construction allows for easy exchange of excitation fibers, which makes it a flexible solution, easily adaptable for different experiments requiring different excitation wavelengths. The concentricity of GGC and EGC was at a level of 20 μm, meaning the excitation fiber (either NCHCF or HC-800-02) and the GRIN lens (GRIN 2908, Thorlabs, Newton, NJ, USA) could have had their main axes shifted by 20 μm. As the GRIN lens outer diameter was 1.8 mm, we were expecting a minimal focal point shift due to this misalignment, and in the case of our experiments it did not pose a problem. The GGC was surrounded by four smaller, symmetrically placed (1 mm diameter) adjacent holes, where the collection fibers (GH-4001 Eska^TM^, Mitsubishi Rayon, Tokyo, Japan) were inserted. These fibers present many advantages in terms of remote fluorescence collection—the large numerical aperture (NA_COL_ = 0.5) and core diameter (Φ_COL_ = 980 μm), combined with good transmission in the visible region [27] make them a suitable choice for remote fluorescence sensing. Although the collection fiber ring was not fully filled because of the collection fiber’s large stiffness, their large NA and core diameter compensated for this loss.

The initial design of this simple probe was imperfect because of large GRIN lens diameter, which resulted in ~1.5 mm offset between the main axes of the collection fiber and GRIN lens. This made the collection fiber’s base aperture too small to collect the fluorescence occurring at the focal point of the GRIN lens. This problem was overcome by polishing the collection fiber’s end-face at a proper angle, which increased the original collection aperture angle. According to Snell’s law, the fiber’s collection cone angle is related to its end-face polishing angle by the following formula:

*β’* = arcsin*(n_col_ ×* sin(*α + γ)) − γ*, (1)

where *β’* is the numerical aperture angle of the collection fiber after polishing, *n_col_* is the collection fiber’s core refraction index, *α* is the maximum angle of refraction at the air–fiber core boundary, with respect to the axis normal to the fibers collection face (in our case—*α* ≈ 19.61°, derived from the Snell’s law), and *γ* is the polishing angle.

**Figure 3:**
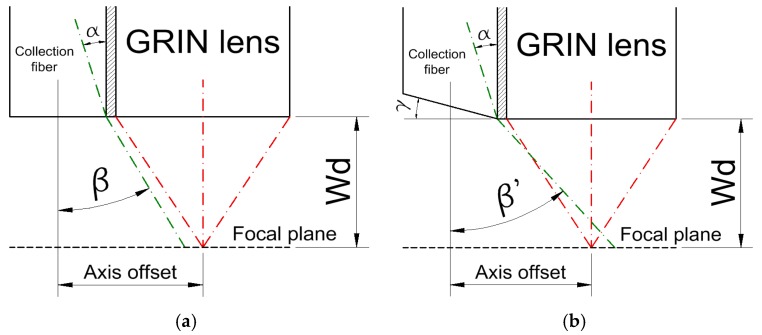
A scheme for the fiber sensor collection tip. In cases of omitting the 15° polish (**a**), the collection fiber’s original aperture angle, *β* (represented as the green dot-dash line), does not cover the GRIN lens focal point, thus virtually no fluorescence signal can be collected. Due to the polishing at the *γ* = 15° angle (**b**), the collection fiber’s acceptance angle changes to *β’*, according to Equation (1). Visible aperture shift occurs, allowing to collect fluorescence signal due to the covering of the GRIN lens focal point. In both pictures, *α* represents the maximum refraction angle at the fiber core-air boundary (approximately 19.61°), while W_d_ stands for the working distance of the GRIN lens. The dimension of the axis offset is approximately 1.5 mm, while W_d_ = 1.449 mm.

One can easily conclude from the equation above that the fiber acceptance cone angle, *β’*, increases with the angle of tip-polishing, *γ*, which also causes the area from which the signal can be collected to rise. According to the above, we have polished the collection faces of each of the collection fibers at the angle of 15°, which was the largest we could afford. Due to such modification, the NA_COL_ changed from 0.5 to 0.68 (the collection fiber’s acceptance angle increased from 30° to 42.8°). The effect of aperture shift before and after the polishing at the chosen angle is presented in Figure 3. The sensor head was made from PMMA (polymethylmethacrylate) to match the collection fibers material, allowing for convenient polishing of the collection fibers tips. The outer diameter of the sensor was 10 mm, which is quite compact and allows it to be used in limited spaces.

## 4. Optical Setup for the Measurements of Multiphoton Fluorescence and Autocorrelation of Ultrashort Laser Pulses

**Figure 4:**
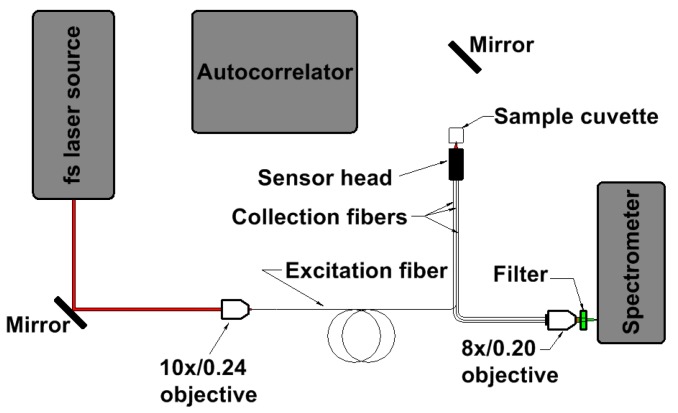
Optical setup schematic. Two mirrors (PF10-03-P01, Thorlabs) were used for the convenience of guiding the laser beam – first one allowed to couple the fundamental beam into the fiber-coupling objective, and the second one was used during the autocorrelation function (ACF) measurements. When measuring the ACF of the setup without the fiber, the GRIN lens was placed directly after the 10× objective, and the beam collimated in this way was directed via the second mirror at the autocorrelator’s aperture. For the ACF measurements of the excitation fibers output beam, the sample cuvette was removed, and the mirror used in the same way as above.

A scheme of the measurement setup is presented in Figure 4. Ultrafast laser pulses, provided by a Ti:Sapphire oscillator (Chameleon Ultra II, Coherent, Santa Clara, CA, USA), were focused by a 10×/0.24 NA microscope objective and coupled into the excitation fiber (HC-800-02 or NCHCF) placed on a 3D translation stage (MBT616D, Thorlabs, Newton, NJ, USA). Coupling efficiencies of ~70% were achieved for both fibers, and an output power of 70 mW (for both excitation fibers) was used during multiphoton fluorescence experiments. Although that may seem relatively high, we did not have to reduce it as no real-life biological samples were investigated. The pulse repetition rate, f_rep_, and output pulse width, τ_pulse_, were 80 MHz and 161.3 fs, respectively. Each of the excitation fibers was 1.5 m long, and each was used for the transmission of different excitation wavelengths—NCHCF for 730 nm and HCPBF for 780 nm. The other end of the excitation fiber was placed in the center of the fiber sensor body. The remaining ends of the collection fibers (fluorescence signal delivery tips) were bundled in a single ferrule, and the fluorescence signal was focused by an 8×/0.2 NA microscope objective on the entrance slit of the spectrometer (S2000, OceanOptics, Dunedin, FL, USA). This 8× objective was additionally mounted on a custom-made, 3D translation stage to ensure proper alignment of the focused signal. Although this spectrometer model is designed for use with optical fibers, this type of setup was necessary for two reasons: firstly, it allowed us to overcome the problem of a large mismatch between the numerical apertures of the spectrometer (NA_spectrometer_ = 0.22) and the collection fibers (NA_COL_ = 0.5) and, secondly, it provided space for the convenient use of optical filters (FESH700, Thorlabs, Newton, NJ, USA), necessary in this epi-fluorescence sensor setup. Initial alignment of the focused signal was performed by coupling a broadband white light source into the collection fibers and finding the position at which the maximum intensity was observed at the spectrometer. After that, a sample cuvette (111-QS, Hellma Analytics, Müllheim, Germany) with the investigated solution was placed in front of the sensor’s face, and its emission spectra were collected. Autocorrelation measurements were performed via an autocorrelator (pulseCheck, A.P.E., Berlin, Germany). Both the spectrometer and the autocorrelator were controlled via a PC. All the data analysis was performed via the MATLAB^®^ custom-written software.

## 5. Fluorescence Measurements

Because the fluorescence was detected in the epi direction, it was necessary to determine the influence that a large amount of reflected light may have on the collected spectra (unwanted emission peaks, spectral shifts, etc.). Two control samples were prepared for this reason—a colloidal silica solution (LUDOX^®^ HS-40, Sigma-Aldrich, St. Louis, MO, USA) and a fluorescein solution (C_fluorescein_ = 10^−5^ M, diluted in 5 × 10^−2^ M NaOH). NADH and FAD (Sigma-Aldrich) were diluted in 10^−2^ M NaOH and H_2_O, respectively. The concentration of each was at the level of 10^−3^ M to compensate for their very low two-photon absorption cross sections [12], which will be further explained in the next section.

## 6. Results and Discussion

### 6.1. Dispersion Effects of Coupling Fundamental Laser Beam into Hollow-Core Fibers

For both excitation fibers, their best wavelength (dispersion- and attenuation-wise) was chosen, resulting in very good performance in terms of temporal pulse broadening. The decision to pick only these two wavelengths was based on the fact that wavelengths of 780 nm and above are widely used for the purpose of multiphoton spectroscopy and microscopy [7,8,9,11], which allows for good imaging results; however, as one can conclude from the work of Denk et al. [1], both the excitation wavelength and pulse duration have a direct influence on the number of photons absorbed during multiphoton absorption process. Thus, it was our goal to show that combining good dispersion parameters and proper excitation wavelength can lead to significantly better fluorescence yields when compared with solutions employing only one of the latter, i.e., fibers transmitting ultrashort pulses with no temporal broadening, but away from the optimal excitation wavelength, or the other way—proper excitation wavelength is at the cost of temporal pulse broadening.

Based on the autocorrelation function (ACF) fitting procedure, the Gaussian signal fit was chosen to estimate the temporal shape of the excitation pulses as presenting the smallest fit root mean square error (RMSE) at the level of 5 × 10^−3^. Thus, the temporal pulse width, *τ_pulse_*, was determined to be (√2)^−1^ × *τ_FWHM_* of the fitted pulses ACF. The values of *τ_pulse_*, obtained for the incident laser and at the outputs of both excitation fibers (730 nm pulses transmitted through the NCHCF and 780 nm pulses transmitted through the HCPBF), were measured to be *τ_pulse_* = 161.3 fs, *τ_NCHCF_* = 162.0 fs, *τ_HCPBF_* = 162.2 fs. The total temporal pulse broadening for the NCHCF and HCPBF was estimated at 0.7 fs and 0.9 fs, respectively, which can be considered negligible, suggesting that both fibers should perform similarly in terms of multiphoton experiments. However, D values of the HC-800-02 increase rapidly for wavelengths below 775 nm, reaching −100 ps/nm × km at 760 nm. The D slope of this fiber at this spectral region can be roughly estimated at the level of 7 ps/nm^2^ × km, which is a few orders of magnitude higher than in the case of the NCHCF (0.01 ps/nm^2^ × km) [18]. As a result of this, the spectral region of 760 nm and below is unavailable for the HCPBF under testing, making it an inferior choice for the purpose of multiphoton excitation of endogenous fluorophores. Additionally, in the case of NCHCF, such a low value of the D slope allows for avoiding higher-order dispersion compensation systems, which simplifies the optical setup.

### 6.2. Two-Photon Induced Fluorescence of Biological Fluorophores Excited with NCHCF and HCPBCF Fibers

**Figure 5:**
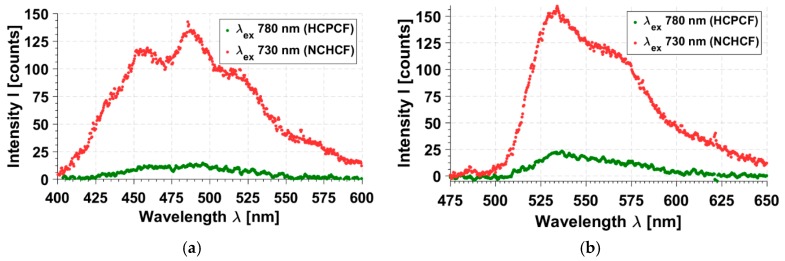
Emission spectra of (**a**) NADH solution in 0.01 M NaOH; and (**b**) FAD solution in water. A visible increase in the total emission signal for both the compounds can be observed for the excitation wavelength of 730 nm. HCPCF stands for Hollow-Core, Photonic Crystal Fiber—HC-800-02 in our case.

Due to the fact that the sensor detected epi-fluorescence from the solution of various fluorophores placed in a quartz cuvette, the light of the fundamental laser beam was partially reflected from the front surface of the cuvette and had high intensity. It was difficult to eliminate it completely, even when using a proper cut-off filter. However, no detector saturation or unexpected emission peaks were observed. The TPEF (two-photon excited fluorescence) spectra of fluorescein, excited at 730 nm and 780 nm, peaked at 520.1 and 519.7 nm, respectively. These results, as well as the observed spectra shapes, are in agreement with literature [28], proving that this setup could be used for fluorescence measurements. The fluorescence emission spectra of the fluorophores of interest, NADH and FAD, are presented in Figure 5.

As was mentioned earlier, both the biological fluorophores had high concentrations of 10^−3^ M. Because of that, a large spectral shift, as well as the overall distortions of both emission spectra, can be observed. Concentrations had to be so high because of very weak two-photon absorption cross sections of both fluorophores at the 780 nm wavelength, making the signal hard to detect in the presented sensor setup, proving the need for its further optimization in terms of the collection efficiency. However, comparing the fluorescence emission intensities for both excitation fibers, HCPBF and NCHCF, one can notice an approximate 6- (FAD) and 9- (NADH) fold increase in the total signal intensity in the case of NCHCF excitation, keeping the power of fundamental beam at the same level. This can be explained by two major factors: firstly, these fluorophores have much larger two-photon absorption cross sections for the 730 nm wavelength, and secondly, the NCHCF fiber allows for temporally and spectrally undistorted transmission of ultrashort pulses of 730 nm. These results prove our initial assumption that fibers combining both good dispersion parameters and transmission bandwidth suitable for the specific fluorophore excitation may become a good choice for non-linear endoscopy devices.

## 7. Conclusions

The application of a new type of fiber—a negative curvature, hollow-core fiber in the multiphoton fluorescence setup—has been presented. A comparison between this fiber and a commercial solution for fiber-based, femtosecond pulse delivery was performed. The total temporal broadening of femtosecond pulses coupled into both fibers was estimated to be at the level of 0.7 fs for the NCHCF fiber at the 730 nm wavelength and 0.9 fs for the HC-800-02 fiber at the 780 nm wavelength. However, the NCHCF has two main advantages over the HCPBF—first, its dispersion-free spectral bandwidth occurs at 680–750 nm, making it a superior solution for the multiphoton fluorescence excitation of endogenous fluorophores. Indeed, the total fluorescence emission signal of NADH and FAD solutions, induced by the 730 nm pulses transmitted through NCHCF, is nearly 6 and 9 times stronger, respectively, when compared to the fluorescence induced with 780 nm pulses transmitted through the commercial HCPBF. Another advantage over the HCPBF is the extremely low D slope of the NCHCF—0.01 ps/nm^2^ × km compared to the 7 ps/nm^2^ × km of HCPBF, which makes it a perfect solution for a fuller utilization of the tunability of current ultrafast laser sources, very important for multiphoton spectroscopy and microscopy. The presented optical fiber sensor still requires improvements in terms of collection efficiency. Its largest drawbacks are the absence of additional fibers in the fiber ring and very large dead volume between the GRIN lens and collection fibers. However, the easily modifiable construction and flexibility provided by the possibility of excitation fiber exchange make the authors believe that this sensor may, eventually, become an interesting alternative to the currently used sophisticated solutions.
